# Three-dimensional laser scanning technology for accurate lower limb edema assessment in heart failure patients: a feasibility study

**DOI:** 10.3389/fcvm.2026.1750128

**Published:** 2026-07-15

**Authors:** Lorenzo Lippi, Mauro Nascimben, Alessio Turco, Fjorelo Refati, Valter De Michelis, Alessandro de Sire, Marco Invernizzi, Lia Rimondini

**Affiliations:** 1 Department of Health Sciences, University of Eastern Piedmont “A. Avogadro”, Novara, Italy; 2 Department of Cardiology, Azienda Ospedaliero-Universitaria SS, Antonio e Biagio e Cesare Arrigo, Alessandria, Italy; 3 Department of Medical and Surgical Sciences, University of Catanzaro “Magna Graecia”, Catanzaro, Italy; 4 Research Center on Musculoskeletal Health, MusculoSkeletalHealth@UMG, University of Catanzaro “Magna Graecia”, Catanzaro, Italy; 5 Translational Medicine, Dipartimento Attività Integrate Ricerca e Innovazione (DAIRI), Azienda Ospedaliero–Universitaria SS, Antonio e Biagio e Cesare Arrigo, Alessandria, Italy

**Keywords:** 3D laser scanner, complete decongestive therapy, heart failure, lower limb edema, lymphovascular invasion

## Abstract

**Introduction:**

Lower limb edema is a common manifestation in older adults with heart failure, adversely affecting both physical well-being and functional capacity. Accurate measurement tools are essential for effective clinical management. This feasibility study aimed to evaluate the potential of three-dimensional laser scanning (3DLS) for lower limb volume assessment by examining its preliminary agreement and repeatability compared the traditional circumferential method (CM).

**Materials and methods:**

Heart failure older adults (*n* = 8; mean age 73 ± 4.51 years) were recruited, and lower limb volumes were assessed with both CM and 3DLS. Different digital volume quantification algorithms were used. Correlation, linear regression, and Bland–Altman plots were used to evaluate the agreement between methods.

**Results:**

Significant correlations were observed between all digital methods and the CM. In particular, DELA VERT-3DLS method showed the highest correlation (r = 0.856; *p* < 0.001). Bland–Altman analysis revealed a consistent trend of overestimation among digital methods.

**Conclusion:**

These preliminary findings suggest that 3DLS is a feasible and potentially practical approach for lower limb volumetric assessment in older adults with heart failure. However, given the small sample size, further studies with larger cohorts are warranted to confirm feasibility and explore reliability, agreement, and clinical applicability.

## 1 Introduction

Lower limb edema, a common manifestation in heart failure patients, results from fluid accumulation in the extremities due to impaired circulation (1). The interplay of factors such as reduced cardiac output, elevated venous pressure, and neurohormonal activation contributes to the development of lower limb edema (2). This condition not only adversely affects patients' physical well-being but also has important implications for their quality of life and healthcare costs (3). Understanding the intricate pathophysiological mechanisms underlying heart failure-related lower limb edema is essential for developing targeted and effective interventions (2, 4).

The management of heart failure-related lower limb edema involves a multifaceted approach aimed at addressing both the underlying cardiac dysfunction and the symptomatic relief of edema (5). Pharmacological interventions, such as diuretics, are commonly prescribed to reduce fluid retention (6, 7). Lifestyle modifications, including sodium restriction, regular exercise, and weight management, play a crucial role in preventing fluid accumulation (8–11). Compression therapy, through the use of compression stockings, aids in improving venous return and minimizing edema (12). The personalized nature of treatment underscores the importance of a comprehensive assessment of each patient's condition to tailor therapeutic strategies for optimal outcomes.

Accurate measurement of lower limb edema is imperative for effective clinical management. Traditional methods, such as the circumferential method (CM) using a tape measure, provide a basic assessment of limb size (13). However, concerns about the reliability and sensitivity of these methods have prompted the exploration of more sophisticated techniques (14). In addition, water displacement (WD) has been proposed, over the last few years, to assess the lower limb volume in the context of peripheral edema (15). Perometry, a volumetric measurement tool, quantifies limb volume changes with greater precision (16). Moreover, bioimpedance analysis assesses tissue impedance to estimate fluid content. Imaging modalities, including ultrasound and magnetic resonance imaging (MRI), offer detailed visualization of tissue fluid distribution (17). Understanding the strengths and limitations of each method is essential for selecting appropriate tools based on the clinical context and the specific needs of heart failure patients with lower limb edema.

Three-dimensional laser scanning (3DLS) is an emerging and innovative technology with strong potential to enhance the assessment of lower limb edema (18, 19). Unlike traditional methods, 3DLS captures the full three-dimensional surface geometry of the limb, offering a comprehensive and precise representation of its volume and shape (19). This non-invasive, contact-free technique provides accurate and highly reproducible measurements (20). Previous comparisons have shown that 3DLS performs as accurately and repeatably as CM and WD for upper limb volume measurements (21, 22). The application of 3DLS in breast cancer-related lymphedema has showed promise in assessing limb volume changes in assessing limb volume changes (23). In the context of heart failure rehabilitation, the use of 3DLS might improve the understanding of lower limb edema dynamics, offering precise data to guide tailored therapeutic interventions (24). Therefore, this feasibility study aimed to explore the potential of 3DLS for lower limb volume measurement in patients with heart failure by examining its preliminary agreement and repeatability compared with the circumferential method (CM).

## 2 Materials and methods

### 2.1 Participants

Older adults with heart failure were recruited between January and May 2023 at the Cardiorespiratory and Post-Operative Cardiac Surgery Rehabilitation Department, Azienda Ospedaliero-Universitaria SS. Antonio e Biagio e Cesare Arrigo, Alessandria, Italy. Inclusion criteria were: (a) age >65 years; (b) confirmed diagnosis of heart failure; and (c) left ventricular ejection fraction <40%. We excluded patients with: a) lymphedema, b) anemia [(Hb) < 9 g/dl]; c) severe thrombocytopenia (<100,000 platelets/mm^3^); d) history of bleeding; e) central nervous system lymphomas; f) presence of medications on the lower limbs; g) Allergy to dermographic marker; h) current infections; i) being unable to sign informed consent. The study was approved by the Institutional Review Board of Alessandria Hospital (PROLI-CAMA; decision no. 0008662, 20th April 2021). Participants were properly informed about the aims of the research, the testing procedures, the treatment of personal data, and the possibility of withdrawal at any time. Written informed consent was obtained from each subject before participating in the experiment, and all procedures were conducted in accordance with the principles of the Declaration of Helsinki.

### 2.2 Outcomes

Each participant underwent both CM and digital measurements during the same session, using the Structure Sensor Mark II laser scanner (3DLS). All measurements were performed by a physical therapist with over 20 years of experience in cardiac rehabilitation.

#### 2.2.1 Circumferential method (CM)

The Circumferential Method (CM) assessment was performed following the guidelines of the International Society of Lymphology, and the protocol was described in the study by Pani et al. (25). The CM consisted of measuring the tape with 1 mm of sensitivity lower limb circumferences of participants. The patient was in a supine position with the lower limb under examination raised, while the operator used a tape to measure the circumference and length.

To minimize measurement error, the lower limb was divided into three segments for volume calculation. The portion from C4 to C7 was considered as a cylinder (cyl-1) with a length of L3 and circumference, obtained from the average of the circumferences from C4 to C7, denoted as Avccyl_1_. The portion from C3 to C4 has been approximated to a cylinder (cyl-2), which measured length L2 and circumference C4. The portion from the toes to the level of C3 was considered to be half the volume of a rectangular cube with L1 and L2 as the length and width. The volumes of the different segments were measured separately using the following formulas (26):

$$V1=\pi \;\times \;(\mathrm{Avccy}l_{1}{/2\pi )}^{2}\;\times \;L3$$

$$V2=\pi \;\times \;{\left(C4/2\pi \right)}^{2}\;\times \;L2$$

V3=(L1×L2×L2)/2

The total volume of the limb is the result of the sum of the volumes found.

#### 2.2.2 Three-dimensional laser scanner (3DLS)

A portable 3DLS system (Structure Sensor Mark II; Occipital Inc., Boulder, CO, USA) attached to an iPad Air 2 (iOS) was used to evaluate lower limb volumes. All data were saved and processed using the software Captevia Rodin4D, Version 3.3.3.1 (Rodin SAS©, Merignac, France). In the CM phase, the circumferences were marked using a dermographic marker to facilitate the acquisition of digital measurements. To guarantee the proper accuracy during the scanning phase, it was necessary to ensure that subjects could maintain a stable position for the entire measurement duration.

Two scanning positions were selected according to each patient's motor abilities and ability to maintain a stable posture:

- Patient with moderate motor impairment: the footboard of the bed was removed and the patient, with the help of the electric bed and the operator, moved to the bottom of the bed to expose the lower limb under examination up to above the knee. From this position, the subject is asked to extend the knee and keep the tibiotarsal joint flexed at 90° for the duration of the measurement, which normally takes about 60 s. The scansion was performed by framing the limb with the sensor from the front, side-right, side-left, top, and bottom at approximately 1 m. The measurement was performed until the affected portion of the limb was captured in its entirety, and no gaps were left in digital reconstruction.
- Patient with slight/no motor impediment: The patient, with the help of an operator or independently, sits on the lateral edge of the bed in favor of the light, exposing the lower limb up to above the knee. Measurement procedures followed the same protocol described above.

As explained in the study by Nascimben et al. (27), the software was developed using Python 3.8. For its implementation, the Open3D and Trimesh libraries were used. The software kit includes three executable applications for Windows, called Edit 3D, Slice 3D, and Cut 3D. Further details on the software used were presented in the previously mentioned study (27).

After scanning, the 3D reconstructed limbs were saved on a laptop and processed offline with the software presented in the previous study (27), using Cut 3D app, which computed all digital volumes under evaluation.

**Figure 1:**
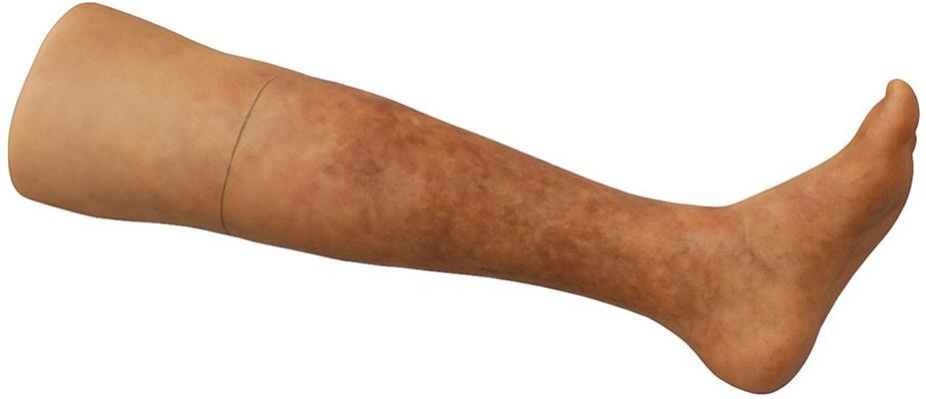
Example of three-dimensional reproduction of a lower limb.

For further details about the 3DLS method, see Figure 1.

### 2.3 Digital volumes employed in the study

The digital nature of the data generated by laser scanners allows for easy visualization, analysis, and manipulation using specialized software: this facilitates detailed inspection, comparison, and documentation of the object's characteristics using multiple computational techniques. The digital volumes enclosed in the study were derived from different digital methodologies. One method involved the so-called “Gift Wrapping” algorithm [digital volume “Exp CH” (28)], which is a convex hull computation used to find the minor convex shape (the “hull”) that encloses a given set of points. In 3D space, the algorithm extends naturally to find the convex hull of a set of points in three dimensions. This algorithm is often used for its simplicity and ease of implementation; however, it might not be efficient for large datasets due to its time complexity, which is typically O(nh), where n is the number of points and h is the number of points on the convex hull. In 3D space, the gift-wrapping algorithm extends the concept of finding the convex hull by considering the points in three dimensions: the idea is to iteratively select the points that form the outermost boundary in the three-dimensional space until a closed convex hull is formed. The alpha shape algorithm is a geometric algorithm used for computing the alpha shape of a set of points in three-dimensional space [volumes “Alpha MED” and “Alpha MEAN” (29)]. The alpha shape represents a generalization of the convex hull and allows the creation of non-convex shapes that enclose a set of points. One crucial aspect is the selection of the parameter “alpha,” which plays a crucial role in determining the alpha shape's level of detail and complexity. Smaller alpha values tend to result in more detailed shapes, including smaller concavities, while larger alpha values lead to simpler, more convex shapes. Selecting an appropriate alpha value is often based on the application's specific requirements. Digital volumes with the “DELA” keyword were based upon the Delaunay triangulation (30), which connects the three-dimensional points, considered as the edges of the leg to be triangulated. Delaunay triangulation involves the creation of tetrahedra (four-faced polyhedra) that connect the input points; this process is often referred to as tetrahedralization. It is an iterative process: the algorithms evaluated all tetrahedra and removed those not satisfying the Delaunay condition; a tetrahedron is Delaunay if and only if no point lies inside the circumsphere of the tetrahedron.

#### 2.3.1 Digital volumes calibration

**Figure 2:**
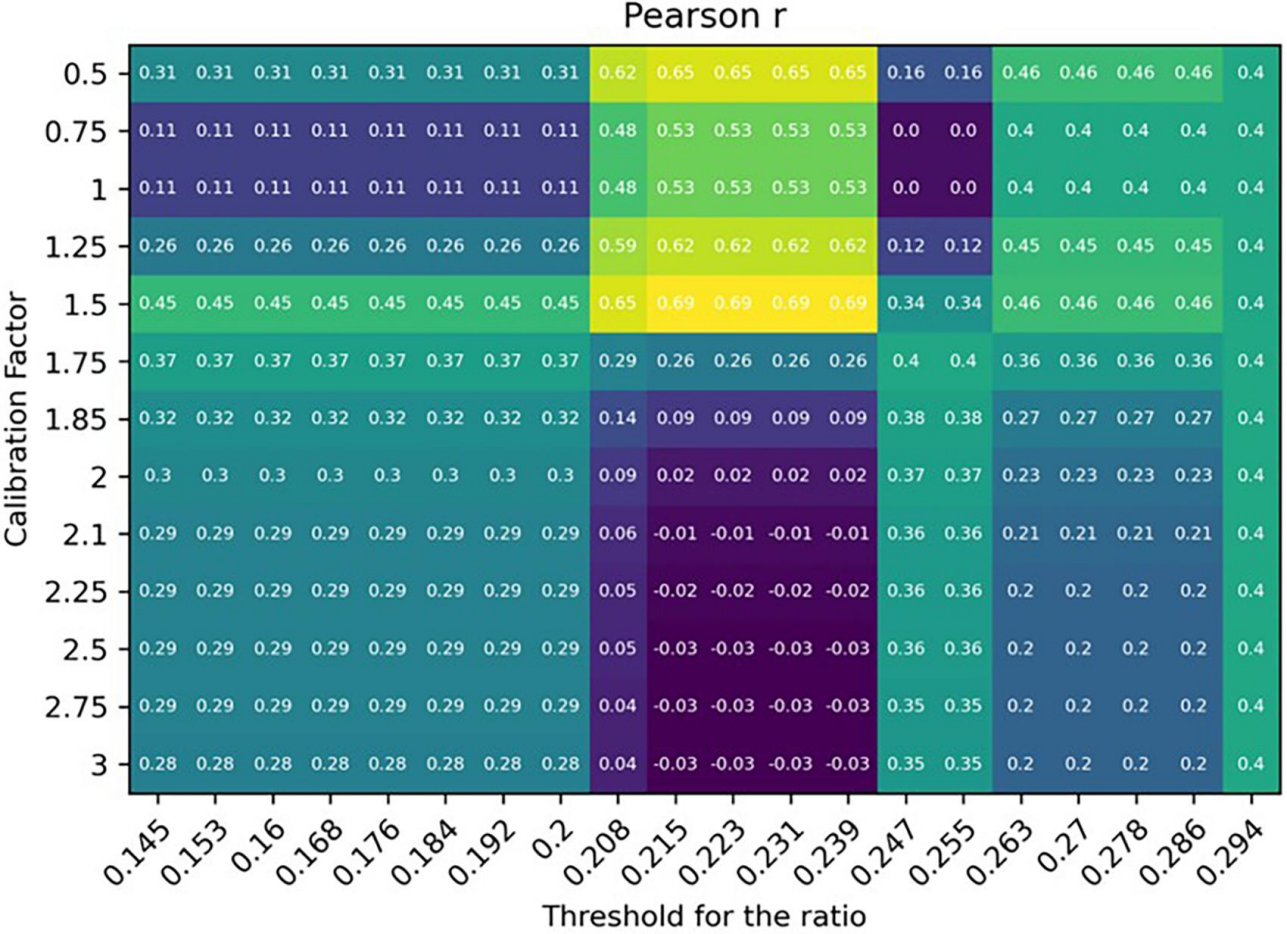
Simulation on healthy subjects: the optimal relationship between digital volume and clinical one requires a correction factor of 1.5 tuned by the digital voxelization ratio.

The calibration procedure was performed to standardize digital reconstruction parameters prior to application in the patient cohort. The digital methods to assess lower limb volumes were initially tested on five healthy volunteers (three men and two women with a mean age of 30.2 ± 2.6 years). In this preliminary study, tiling algorithms were also employed: they convert a continuous geometric shape or object into a grid of discrete volumetric pixels, known as voxels (31). This grid representation allows for calculating 3D shape volumes by counting the number of voxels occupied by the object. The final volume was calculated based on the voxel count and the volume of an individual voxel. The procedure was successfully tested in (32) to evaluate the tortuosities of the hand, leading to an updated version of Cut 3D app. However, this methodology is parameter-specific: the edge of the voxel influences volume accuracy. During this preliminary study, it has been noticed that when the ratio between the volume computed by voxelization (creating a cube around each vertex of the mesh) with cube side length as in (32) (called “TASS 1”), and the volume calculated with a resolution equal to the mean distance between nearest neighbor in the 3D point cloud was above a threshold, the digital volumes tend to diverge from the clinical measurements. A simulation on the healthy volunteers' leg volumes has been conducted to evaluate the optimal range of values leading to improved agreement in the preliminary simulation setting (Figure 2). The Pearson correlation between expected volume and digital counterparts was maximal when the ratio between voxelization volumes was in a range between 0.208 and 0.239, applying a correction factor of 1.5. For this reason, this value has been introduced to adjust the leg's digital volume estimation on patients, when the “TASS 1” vs. “TASS 3” ratio was deviating from expected as observed on healthy volunteers (Supplementary Table S1). This algorithmic check was fully automated within the software architecture, ensuring that the same mathematical criteria were objectively applied across all scans. The implementation of this correction factor is further supported by the Bland–Altman analysis, which identified a constant, additive pattern of systematic overestimation rather than a proportional error, making a standardized offset adjustment appropriate for maintaining internal consistency.

**Figure 3:**
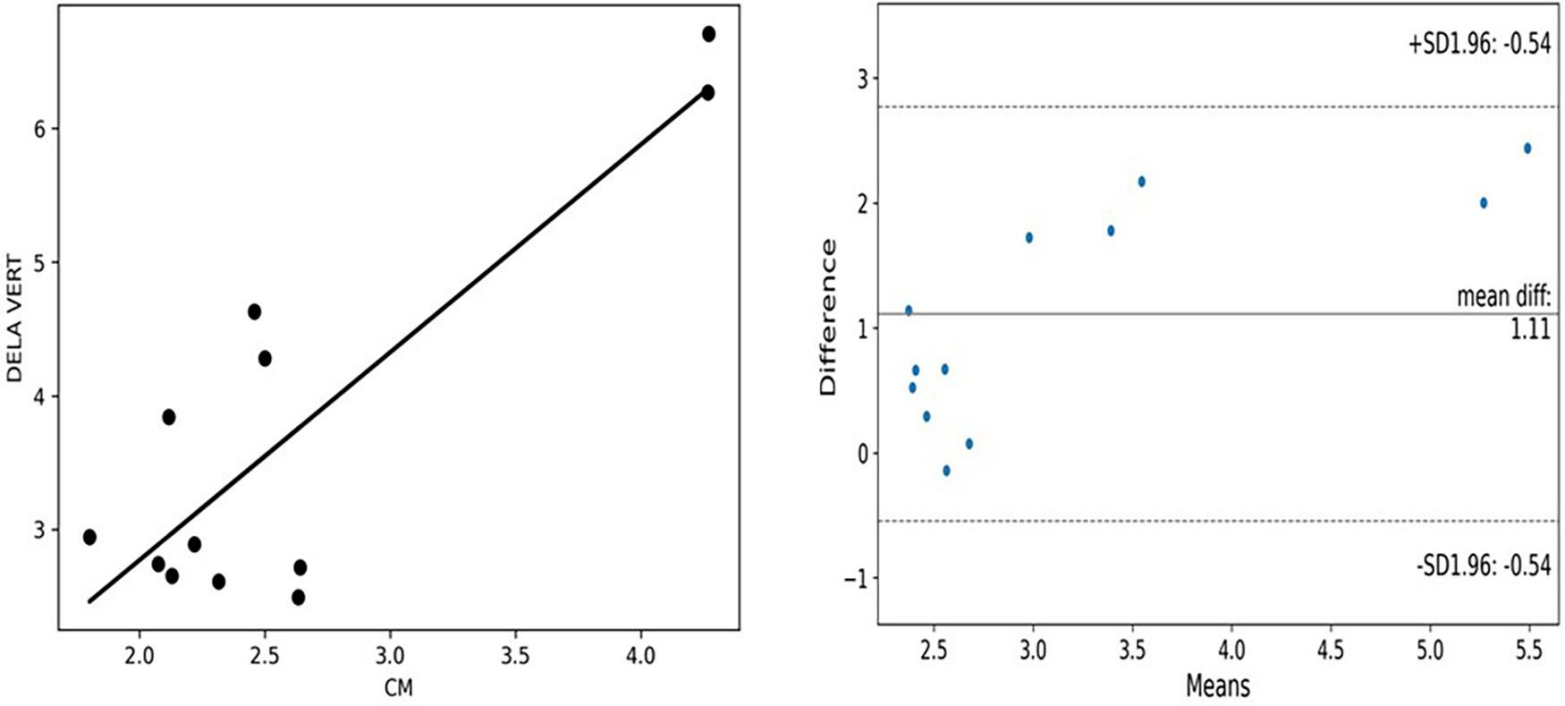
On the left: linear regression between the CM and DELA VERT method. On the right: Bland–Altman plot between the CM and DELA VERT digital method.

The ratio between tiling volumes provided a descriptor of digital volumes' behavior: when the ratio is inside a certain range, the volumetry looks accurate, whereas outside a certain range, it requires the introduction of a correction factor to maintain internal consistency of the digital volume estimation process. Additional remarks on the voxelization procedure were included in the Supplementary Materials. The resulting control sequence based on the ratio between the voxelization volumes is exemplified in Figure 3 and implemented to adjust digital volumes outcomes.

**Figure 4:**
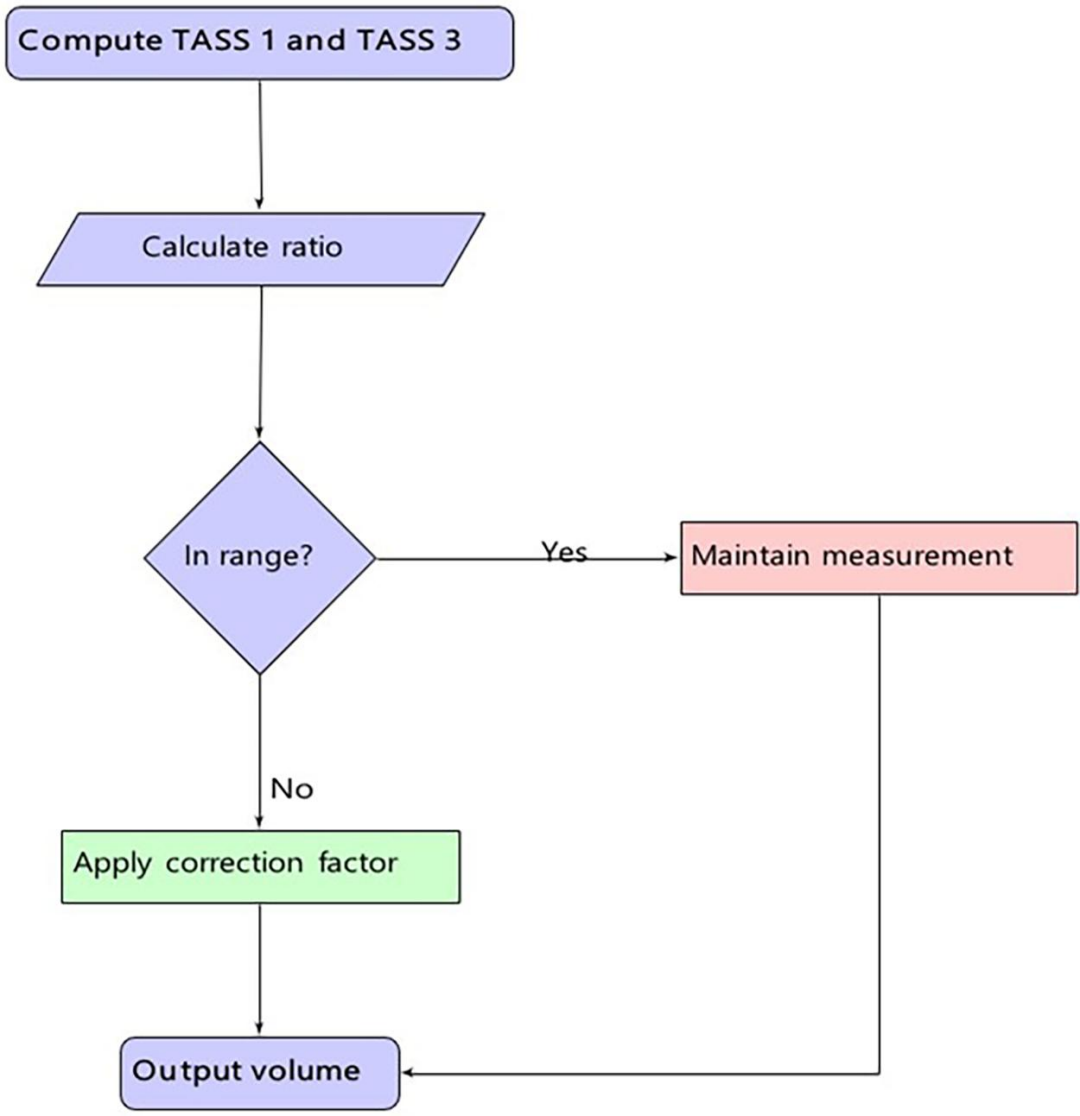
Control sequence employed to adjust the digital volumes. The sequence controls if the ratio between voxelization volumes exceeds a certain value: in that case, a correction factor is applied to avoid overestimation.

It should be noted that the calibration procedure and correction factor were derived from a small sample of healthy young volunteers and were intended to optimize the technical performance of the digital reconstruction algorithms. This calibration was not specifically validated in edematous older adults with heart failure. Therefore, the applicability of the correction factor in this clinical population should be interpreted with caution, and further validation studies in disease-specific cohorts are warranted.

### 2.4 Statistical analysis

Statistical analyses were performed using GraphPad Prism version 6.0 (GraphPad Software, Inc., San Diego, CA, USA). The association between lower limb volumes measured with the circumferential method (CM) and 3DLS was assessed using Pearson's correlation coefficient (r), with corresponding *p*-values reported. Formal intra- and inter-rater reliability analyses were not conducted in this feasibility study.

Agreement between methods was evaluated using Bland–Altman analysis. Bias was calculated as the mean difference between 3DLS and CM measurements and is reported in dm^3^. A positive bias indicates overestimation by 3DLS relative to CM, whereas a negative bias indicates underestimation. The 95% limits of agreement (mean difference ± 1.96 SD) were calculated to assess the dispersion of differences between methods.

## 3 Results

Of 12 patients screened for eligibility, 8 met the inclusion criteria and were enrolled in the study. Four patients declined to participate, resulting in a final sample of eight subjects. Five participants were male (62.5%), and 3 were female (37.5%). The age of the participants recruited ranged between 66 and 80 years (mean age 73 ± 4.51 years). A total of 12 lower limbs were analyzed; the left limbs of three participants were excluded due to non-compliance with the inclusion criteria. Descriptive statistics of the different 3DLS methodologies were reported in Table 1.

**Table 1:** Descriptive statistics of the different 3DLS methodologies: data summary (in dm^3^).

Method	Mean	Median	Standard deviation	Min	25%	75%	Max
Exp CH	4.97	4.38	2.08	2.60	3.02	6.20	8.81
Alpha MED	4.86	4.33	1.98	2.55	3.02	6.02	8.42
Alpha MEAN	4.78	4.25	1.95	2.53	2.95	5.93	8.26
DELA VERT	4.73	4.20	1.94	2.49	2.93	5.87	8.21
DELA PCD	4.69	4.16	1.94	2.46	2.90	5.81	8.14
CM	2.62	2.39	0.81	1.80	2.13	2.64	4.27

Min, minimum value; 25%, 25th percentile; 75%, 75th percentile; Max, maximum value.

The statistical analysis highlighted a significant correlation between all digital methods and the clinical method. In particular, the method that best expresses the correlation with CM was the DELA VERT (r = 0.856; *p*-value = 0.0049). Other significant correlations were observed in Exp CH (r = 0.847, *p* = 0.0005), Alpha MED (r = 0.851, *p* = 0.00045), Alpha MEAN (r = 0.852, *p* = 0.00043), and DELA PCD (r = 0.851, *p* = 0.00044). These results indicate a moderate positive relationship between digital and circumferential measurements (Table 2).

**Table 2:** Correlation between digital volumes and circumference measurements.

Method	r	r^2^	*p*-value
Exp CH	0.847	0.718	0.00050
Alpha MED	0.851	0.724	0.00045
Alpha MEAN	0.852	0.726	0.00043
DELA VERT	0.856	0.733	0.00038
DELA PCD	0.851	0.725	0.00044

r, Pearson coefficient; r^2^, coefficient of determination.

The analysis conducted according to the Bland–Altman method highlighted that all the digital volumes considered fall within the ranges of concordance. Positive BIAS values were observed for all digital methods, indicating a consistent trend of overestimation. More in detail, digital volumes based on Delaunay triangulation exhibited the smallest BIAS compared to the reference standard. The detailed results for mean bias, standard deviations, and 95% limits of agreement for each computational method were summarized in Table 3.

**Table 3:** Bland–Altman: bias and limits of agreement.

Method	BIAS	SD of Bias	Lower LOA SD	Upper LOA SD
Exp CH	1.356	0.969	−0.543	3.255
Alpha MED	1.242	0.882	−0.488	2.971
Alpha MEAN	1.162	0.854	−0.513	2.836
DELA VERT	1.113	0.845	−0.544	2.77
DELA PCD	1.073	0.851	−0.596	2.742

Bias, mean difference. Positive bias indicates 3DLS overestimates CM, negative bias indicates underestimation. LOA, 95% limits of agreement (bias ± 1.96 × SD). SD, standard deviation of the differences. Units, dm^3^.

## 4 Discussion

Given the exploratory design and limited sample size, the findings should be interpreted as preliminary. The significant correlation observed between all digital and clinical methods, particularly the DELA VERT algorithm, highlights the potential of digital techniques for volumetric assessment. Bland–Altman analysis revealed a consistent trend of positive mean bias values among all digital methods, indicating that 3DLS systematically yielded higher volume values compared with the circumferential method (overestimation). Although correlation was observed, correlation alone does not imply agreement, and this systematic bias must be taken into account when interpreting measurement comparability. Further studies investigating the sources of this bias could help clarify sources of measurement variability and optimize digital volumetric tools. For example, Mestre et al. (33) reported a similar overestimation trend between water displacement (WD) and 3DLS-derived digital volumes. Our findings of systematic overestimation are consistent with this study, which also observed a positive bias when comparing 3DLS-derived digital volumes with water displacement methods. These consistent trends suggest that 3DLS may provide a broader volumetric assessment that includes subtle surface geometries often missed by manual tape measurements. This observation is consistent with previous studies conducted on the upper limb (27) and the hand (32) using the same technology, which also reported a tendency toward overestimation. In a larger cohort, the authors reported evidence supporting the validity of lower limb segmental volumetry by comparing real-time 3D reconstruction with the WD method. The authors evaluated patients with chronic venous insufficiency, patients with lymphedema, and healthy controls, reporting that 3DLS suggested accuracy and reproducibility and appeared suitable for limb volume evaluation. Another study reported significant results in measuring lower limb volume (34): the authors included 30 patients with chronic venous disease and performed a comparison between the 3DLS method and the CM method. They found that the 3DLS method and the CM showed a significant correlation (r^2^ = 0.9065), and high intraoperator and interoperator reliability were reported through the 3DLS. The manuscript of Cau et al. (21) highlighted the applicability of digital methods in lower limb volumetric quantification, evaluating normal subjects (r^2^ = 0.83). The observed correlations with the circumferential method suggest that digital techniques may represent a promising area for further investigation in clinical conditions involving limb volume changes.

Although most current literature focuses on upper limb volumetry in conditions such as breast cancer-related lymphedema, our study addresses the existing knowledge gap regarding lower limb assessment. Extending volumetric assessment research to the lower limb, especially in patients with heart failure, represents an initial exploratory contribution to the field. Indeed, the systematic review conducted by Bahadori et al. (19) highlighted the lack of comparative studies focusing on the lower limb. By adopting a hand-held capturing device and ad-hoc software solution, we aimed to contribute specific data on the lower limb volumetrics, particularly in patients with heart failure. This broadens the understanding of digital volumetric tools within clinical practice and highlights their applicability across diverse patient populations.

A recent study conducted by (24) explored the reliability of a portable 3D scanner for volumetric quantification of the ankle and foot, providing valuable insights into the potential applications of digital methods in lower limb assessments. Despite the study's focus on healthy subjects, the significant correlation (r = 0.93) emphasizes the potential of these tools in capturing volumetric changes. However, the contextual limitation of investigating only the ankle and foot in healthy subjects underscores the need for further exploration in complex clinical scenarios such as heart failure or lymphedema in the lower limb.

Our study highlights the possible implications of 3DLS and suggest potential practical advantages of 3DLS compared to other methods. In particular, laser scanners can capture detailed geometric information rapidly (35). They can generate detailed 3D models quickly, whereas the process of water displacement can be time-consuming, which involves filling a container with water, submerging the object, and measuring the displaced water volume. The same applies to manual circumferential measurements used in healthcare settings, which require taking circumferential measurements at regular intervals along the limb; these measurements are then used to calculate the overall volume. Another advantage of 3DLS measurements in clinical settings is that they can be considered non-contact methods, allowing for measurements without physically touching the patient. Additionally, 3DLS produces a comprehensive representation of the limbs' surfaces (36): this allows for a more thorough analysis of the limb's shape and dimensions than manual circumferential measurements, which might be limited to returning the approximated total volume and area of limb slices at specific points or sections. Also, manual circumferential measurements may be prone to variations in technique and human error, leading to less consistent results.

Lastly, objective data are crucial in the implementation of artificial intelligence tools aiming at improving patients' precise assessment, including data about clinical characteristics, in order to achieve personalized treatments in clinical settings.

Although this exploratory study suggests potential advantages of 3DLS for lower limb volumetric quantification, several limitations must be acknowledged. First, the sample size was limited, reflecting the exploratory and feasibility-oriented design. Although statistically significant correlations and agreement were observed, larger studies are needed to support these findings and better define the measurement properties of 3D laser scanning in this population. Second, the circumferential method (CM) was used as a practical clinical comparator rather than a gold-standard reference. While CM is widely used in routine care, it has inherent operator-dependent variability. Therefore, agreement with CM should be interpreted as comparative performance against standard clinical practice, not formal validation against a gold-standard technique. In addition, the calibration procedure applied to optimize digital volume reconstruction was derived from a small cohort of healthy volunteers. Although intended to standardize the technical performance of the algorithms, this correction factor was not specifically validated in edematous older adults with heart failure. Furthermore, technical aspects such as the patient's ability to maintain a perfectly stable posture during the 60-second scanning window and the manual placement of dermographic markers could introduce subtle operational variations. Finally, formal intra- and inter-rater reliability analyses were not performed in this preliminary trial, which limits our ability to quantify operator-dependent variance. Consequently, future studies must prioritize large-scale validation protocols that include repeated-measures designs to formally establish the intra- and inter-operator reliability of 3DLS in clinical settings. Future studies should include larger and more diverse samples, as well as comparative evaluations with alternative technologies such as perometry, bioimpedance, and high-frequency ultrasound, and should assess disease-specific calibration parameters to further strengthen the robustness of digital volumetry in clinical settings. Such comprehensive assessments will contribute to a more nuanced understanding of the strengths and limitations of various volumetric measurement tools and their potential applications in clinical and rehabilitation settings. As digital and augmented reality technologies continue to evolve in clinical diagnostics and patient care (37), adapting 3DLS for lower limb evaluation could facilitate its integration into routine clinical workflows.

## 5 Conclusions

In this exploratory study, 3D laser scanning–based digital volumetry showed significant correlations and preliminary agreement with the circumferential method in older adults with heart failure. Among the tested computational approaches, Delaunay-based reconstruction exhibited the most consistent performance relative to the clinical comparator. While these findings suggest that digital volumetry may represent a feasible and promising tool for limb volume assessment, further validation in larger and disease-specific cohorts is required before definitive conclusions regarding clinical applicability can be drawn.

## Data Availability

The raw data supporting the conclusions of this article will be made available by the authors, without undue reservation.

## References

[1] Kakouros NS Kakouros SN. Clinical assessment in acute heart failure. Hellenic J Cardiol. (2015) 56(4):285–301. 26233768

[2] Abassi Z Khoury EE Karram T Aronson D. Edema formation in congestive heart failure and the underlying mechanisms. Front Cardiovasc Med. (2022) 9:933215. 10.3389/fcvm.2022.933215 36237903 PMC9553007

[3] Franks PJ Quéré I Keeley V Tilley A Liebl M Murray S. Quality of life and costs within decongestive lymphatic therapy in patients with leg lymphedema: a multicountry, open-label, prospective study. Lymphat Res Biol. (2021) 19(5):423–30. 10.1089/lrb.2021.0057 34582725

[4] Breidthardt T Irfan A Klima T Drexler B Balmelli C Arenja N. Pathophysiology of lower extremity edema in acute heart failure revisited. Am J Med. (2012) 125(11):1124.e1–e8. 10.1016/j.amjmed.2011.12.015 22921885

[5] Tiwary SK Katiyar VK. Overview of management in lower limb edema. In: Approach to Lower Limb Oedema. Singapore: Springer Nature Singapore (2022). p. 269–84.

[6] Felker GM Ellison DH Mullens W Cox ZL Testani JM. Diuretic therapy for patients with heart failure: JACC state-of-the-art review. J Am Coll Cardiol. (2020) 75(10):1178–95. 10.1016/j.jacc.2019.12.059 32164892

[7] Oh SW Han SY. Loop diuretics in clinical practice. Electrolyte Blood Press. (2015) 13(1):17–21. 10.5049/ebp.2015.13.1.17 26240596 PMC4520883

[8] Clark AL Cleland JG. Causes and treatment of oedema in patients with heart failure. Nat Rev Cardiol. (2013) 10(3):156–70. 10.1038/nrcardio.2012.191 23319101

[9] Doukky R Avery E Mangla A Collado FM Ibrahim Z Poulin MF. Impact of dietary sodium restriction on heart failure outcomes. JACC Heart Failure. (2016) 4(1):24–35. 10.1016/j.jchf.2015.08.007 26738949 PMC4705447

[10] Ratchford EV Evans NS. Approach to lower extremity edema. Curr Treat Options Cardiovasc Med. (2017) 19(3):16. 10.1007/s11936-017-0518-6 28290004

[11] Bozkurt B Fonarow GC Goldberg LR Guglin M Josephson RA Forman DE. Cardiac rehabilitation for patients with heart failure: JACC expert panel. J Am Coll Cardiol. (2021) 77(11):1454–69. 10.1016/j.jacc.2021.01.030 33736829

[12] Urbanek T Juśko M Kuczmik WB. Compression therapy for leg oedema in patients with heart failure. ESC Heart Failure. (2020) 7(5):2012–20. 10.1002/ehf2.12848 32710511 PMC7524111

[13] te Slaa A Mulder P Dolmans D Castenmiller P Ho G van der Laan L. Reliability and reproducibility of a clinical application of a simple technique for repeated circumferential leg measurements. Phlebology. (2011) 26(1):14–9. 10.1258/phleb.2009.009073 20881309

[14] Houwen F Stemkens J de Schipper PJ van der Wouw P Heitink M van Langen H. Estimates for assessment of lymphedema: reliability and validity of extremity measurements. Lymphat Res Biol. (2022) 20(1):48–52. 10.1089/lrb.2019.0082 33751914 PMC8892971

[15] Brodovicz KG McNaughton K Uemura N Meininger G Girman CJ Yale SH. Reliability and feasibility of methods to quantitatively assess peripheral edema. Clin Med Res. (2009) 7(1-2):21–31. 10.3121/cmr.2009.819 19251582 PMC2705274

[16] Sharkey AR King SW Kuo RY Bickerton SB Ramsden AJ Furniss D. Measuring limb volume: accuracy and reliability of tape measurement versus perometer measurement. Lymphat Res Biol. (2018) 16(2):182–6. 10.1089/lrb.2017.0039 28956715

[17] Gasparis AP Kim PS Dean SM Khilnani NM Labropoulos N. Diagnostic approach to lower limb edema. Phlebology. (2020) 35(9):650–5. 10.1177/0268355520938283 32631171 PMC7536506

[18] Hayn D Fruhwald F Riedel A Falgenhauer M Schreier G. Leg edema quantification for heart failure patients via 3D imaging. Sensors (Basel, Switzerland). (2013) 13(8):10584–98. 10.3390/s130810584 23948874 PMC3812619

[19] Bahadori S Immins T Wainwright TW. Volumetric assessment of lower limb oedema using 3D Laser scanning technique: a systematic review. J Med Eng Technol. (2022) 46(1):40–5. 10.1080/03091902.2021.1970841 34647841

[20] Lippi L Turco A Moalli S Nascimben M Curci C de Sire A. Quantitative assessment of upper-limb volume: implications for lymphedema rehabilitation? Appl Sci. (2023) 13(17):9810. 10.3390/app13179810

[21] Cau N Galli M Cimolin V Aranci M Caraceni A Balzarini A. Comparative study between circumferential method and laser scanner 3D method for the evaluation of arm volume in healthy subjects. J Vasc Surg Venous Lymphat Disord. (2016) 4(1):64–72. 10.1016/j.jvsv.2015.05.005 26946898

[22] McKinnon JG Wong V Temple WJ Galbraith C Ferry P Clynch GS. Measurement of limb volume: laser scanning versus volume displacement. J Surg Oncol. (2007) 96(5):381–8. 10.1002/jso.20790 17477361

[23] de Sire A Losco L Cigna E Lippi L Gimigliano F Gennari A. Three-dimensional laser scanning as a reliable and reproducible diagnostic tool in breast cancer related lymphedema rehabilitation: a proof-of-principle study. Eur Rev Med Pharmacol Sci. (2020) 24(8):4476–85. 10.26355/eurrev_202004_21030 32373985

[24] Beldame J Sacco R Munoz MA Masse M Lalevée M. Assessment of the efficiency of measuring foot and ankle edema with a 3D portable scanner. Bioengineering (Basel, Switzerland). (2023) 10(5):549. 10.3390/bioengineering10050549 37237619 PMC10215399

[25] Pani SP Vanamail P Yuvaraj J. Limb circumference measurement for recording edema volume in patients with filarial lymphedema. Lymphology. (1995) 28(2):57–63. 7564492

[26] Sitzia J. Volume measurement in lymphoedema treatment: examination of formulae. Eur J Cancer Care (Engl). (1995) 4(1):11–6. 10.1111/j.1365-2354.1995.tb00047.x 7620649

[27] Nascimben M Lippi L Fusco N Invernizzi M Rimondini L. A software suite for limb volume analysis applicable in clinical settings: upper limb quantification. Front Bioeng Biotechnol. (2022) 10:863689. 10.3389/fbioe.2022.863689 36798789 PMC9928154

[28] Avis D Bremner D. How good are convex hull algorithms? In: Proceedings of the Eleventh Annual Symposium on Computational Geometry; Vancouver, British Columbia, Canada. New York, NY, USA: Association for Computing Machinery (1995). p. 20–8.

[29] Ohbuchi R Takei T. Shape similarity comparison of 3D models using alpha shapes. In: 11th Pacific Conference on Computer Graphics and Applications; 2003; Proceedings. *IEEE* (2003). p. 293–302.

[30] Musin OR. Properties of the Delaunay triangulation. In: Proceedings of the Thirteenth Annual Symposium on Computational Geometry; Nice, France. New York, NY, USA: Association for Computing Machinery (1997). p. 424–6.

[31] Wang SW Kaufman AE. Volume-sampled 3D modeling. IEEE Comput Graph Appl. (1994) 14(5):26–32. 10.1109/38.310721

[32] Nascimben M Lippi L Fusco N De Sire A Invernizzi M Rimondini L. Technical aspects and validation of custom digital algorithms for hand volumetry. Technol Health Care. (2023) 31(5):1835–54. 10.3233/THC-220694 37302048 PMC10578236

[33] Mestre S Veye F Perez-Martin A Behar T Triboulet J Berron N. Validation of lower limb segmental volumetry with hand-held, self-positioning three-dimensional laser scanner against water displacement. J Vasc Surg Venous Lymphat Disord. (2014) 2(1):39–45. 10.1016/j.jvsv.2013.08.002 26992967

[34] Yang WT Zheng K Ren HL Wang SX Sun MS Gong C. Three-dimensional laser scanner as a new tool for measuring lower limb volume in patients with chronic venous diseases. J Vasc Surg Venous Lymphat Disord. (2023) 11(1):127–35. 10.1016/j.jvsv.2022.06.010 35940450

[35] Ebrahim MA-B. 3D laser scanners’ techniques overview. Int J Sci Res. (2015) 4(10):323–31. 10.21275/SUB158346

[36] Karatas OH Toy E. Three-dimensional imaging techniques: a literature review. Eur J Dent. (2014) 8(1):132–40. 10.4103/1305-7456.126269 24966761 PMC4054026

[37] Invernizzi M Runza L De Sire A Lippi L Blundo C Gambini D. Integrating augmented reality tools in breast cancer related lymphedema prognostication and diagnosis. J Vis Exp. (2020) (156):e60093. 10.3791/60093 32090996

