# Supplementary material for: Three-dimensional laser scanning technology for accurate lower limb edema assessment in heart failure patients: a feasibility study
Source: Front Cardiovasc Med. 2026 Jul 15;13:1750128. doi: 10.3389/fcvm.2026.1750128 (PMC13415577; doi:10.3389/fcvm.2026.1750128)
Supplement: Supplementary file 1 [file Datasheet1.docx]

Supplementary Material

# Supplementary Data

## Voxelization for Volume Calculation

To address volumetry using voxelization (also called tessellation or tiling), one should determine the resolution of the voxel grid. The resolution defines the size of the voxels and influences the level of detail in the voxelized representation; higher resolution results in more accurate but computationally expensive voxelization. Other than the grid definition, the user should also decide on an appropriate voxelization algorithm based on the geometry of the object. Common voxelization algorithms include: rasterization-based methods, distance field-based methods, or octree-based methods. The latter employs an octree data structure to represent efficiently and voxelized complex shapes, whereas rasterization converts geometric primitives (triangles, lines, etc.) into matrices. The Distance Field-based methods use distance fields to represent the shape and convert it into a voxel grid. Additionally, implement the chosen voxelization algorithm to convert the 3D shape into a voxel grid. This involves determining which voxels are inside or outside the object based on the original shape’s geometry. For all these reasons, being voxelization a user-dependent and parameter-dependent procedure, it has not been included for patient volume assessment. However, during the current investigation, voxelization volumes proved to be efficient because their ratio acted as a control mechanism to adjust digital volume outcomes.

# Supplementary Tables

The table reports the digital volumes computed on the healthy subjects and the “Ratio” between “TASS 1” and “TASS 3” volumes. Voxelization-derived volumes were not accurate due to the parameter-dependent outcomes they produced. However, they proved to be efficient in providing a control mechanism to detect when digital volumes were deviating from the expected.

**Supplementary Table S1**. Volumes on healthy subjects.

| **Subject** | **Side** | **Exp CH** | **Alpha MED** | **Alpha**  **MEAN** | **DELA VERT** | **DELA PCD** | **Ratio** |
| --- | --- | --- | --- | --- | --- | --- | --- |
| Subj 1 | R | 4.482067 | 4.196669 | 3.952185 | 3.790778 | 3.507229 | 0.153091 |
| Subj 1 | L | 3.805049 | 3.471708 | 3.288074 | 3.146254 | 2.887974 | 0.152467 |
| Subj 2 | R | 5.663103 | 5.219497 | 5.015013 | 4.819106 | 4.414766 | 0.082422 |
| Subj 2 | L | 5.939435 | 5.801989 | 5.532959 | 5.331015 | 5.226960 | 0.152570 |
| Subj 3 | R | 3.604168 | 3.263990 | 3.219906 | 2.975943 | 2.877297 | 0.178016 |
| Subj 3 | L | 3.661736 | 3.301116 | 3.106304 | 3.033410 | 2.839394 | 0.237489 |
| Subj 4 | R | 5.089779 | 4.743681 | 4.610351 | 4.387912 | 4.237473 | 0.154556 |
| Subj 4 | L | 5.059955 | 4.793462 | 4.474784 | 4.447802 | 4.167650 | 0.197864 |
| Subj 5 | R | 5.623424 | 5.488342 | 5.032681 | 4.936321 | 4.680913 | 0.142520 |
| Subj 5 | L | 5.195931 | 4.918478 | 4.637839 | 4.458455 | 4.452531 | 0.158520 |

* Under column Side the letter R is for Right, whereas L stands for Left.

# Supplementary Figures


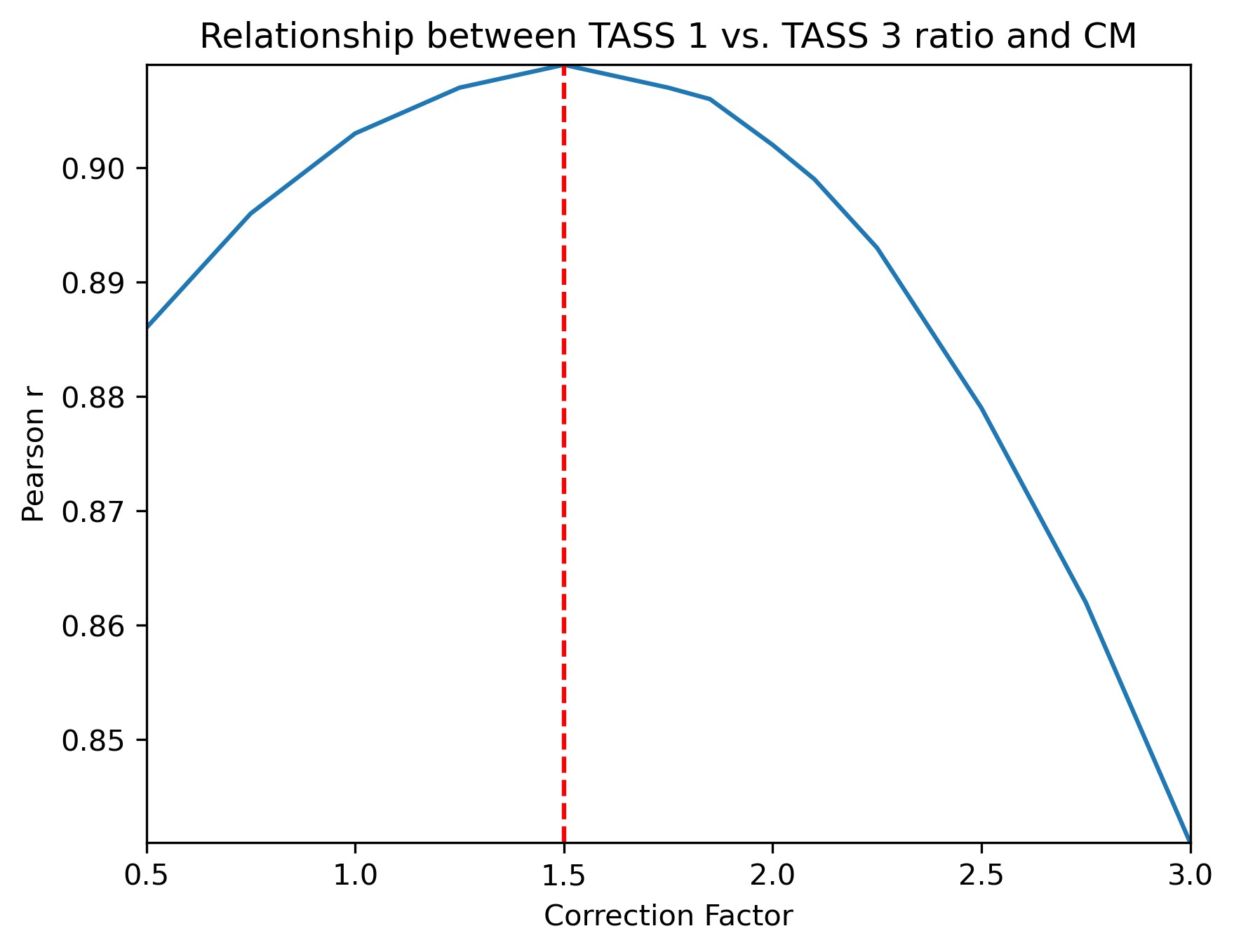


**Supplementary Figure 1.** Relation between voxelizatrion ratio and correction factor. Pearson r modification when adjusting the correction factor. As found in healthy subjects, the 1.5 correction factor leads to maximal correlation between the digital ratio of TASS1 vs. TASS 3 and CM.


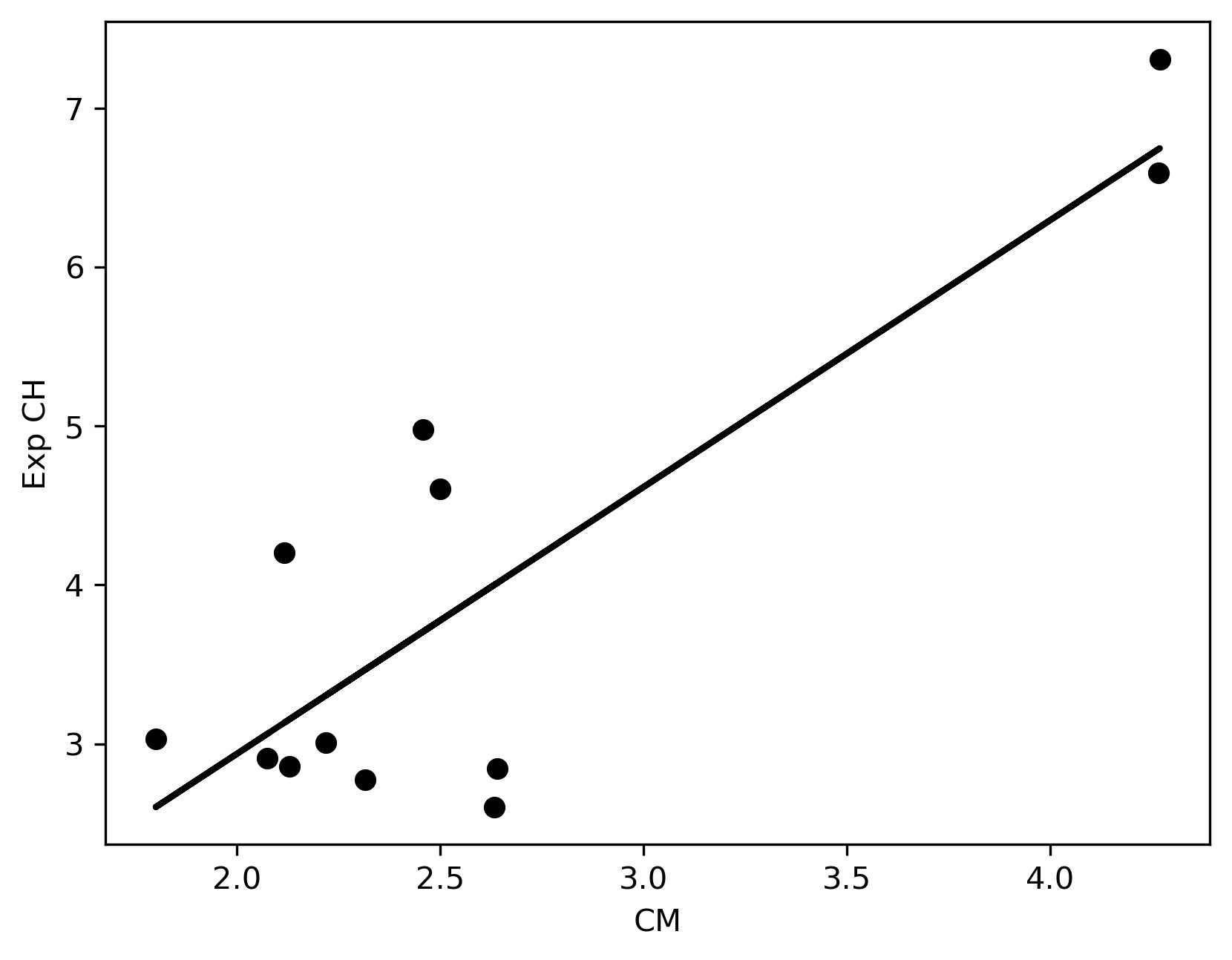

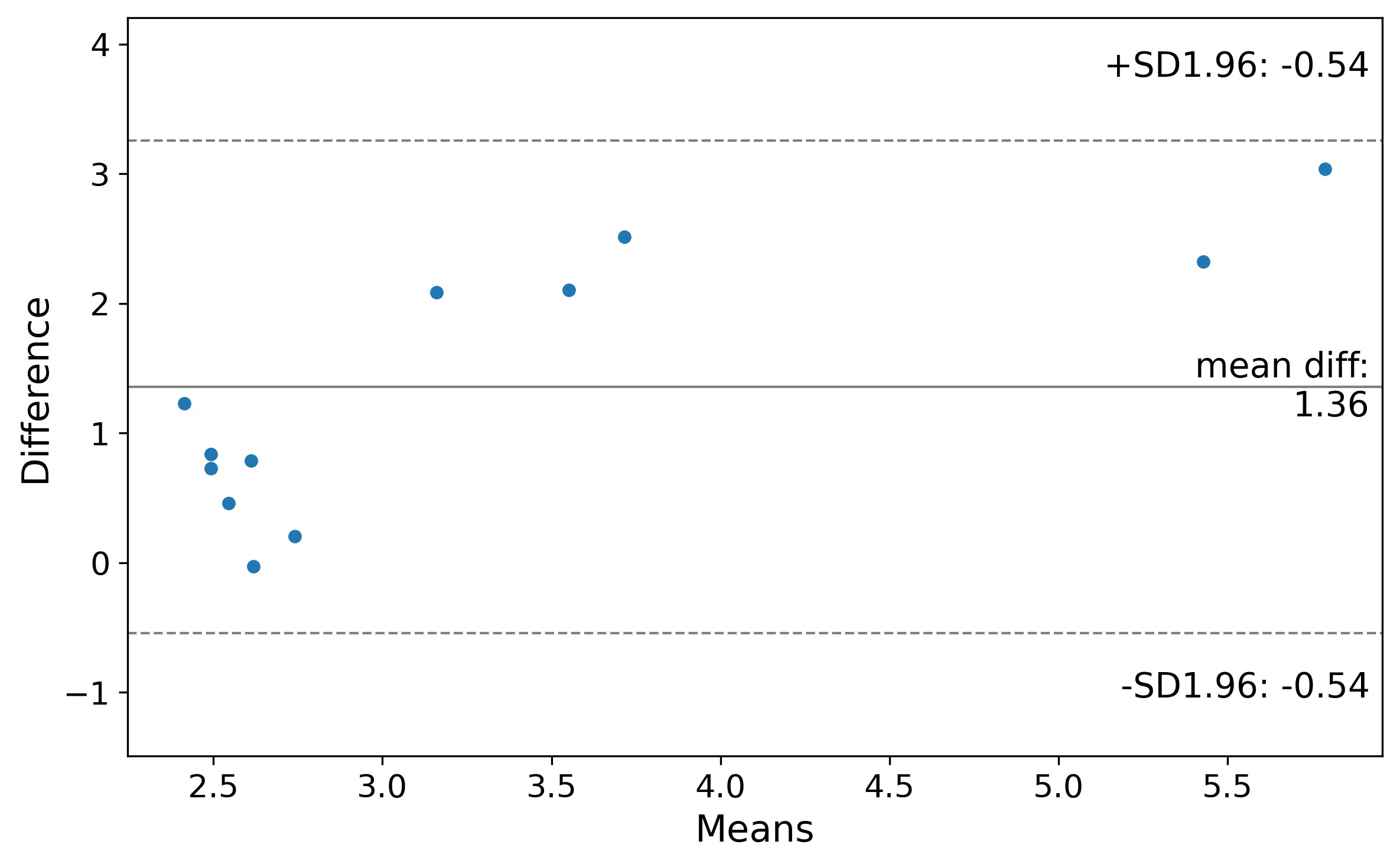


**Supplementary Figure 2.** Bland-Altman and regression plot. On the left: Linear regression between the CM and Exp CH method. On the right: Bland-Altman plot between the CM and Exp CH digital method.


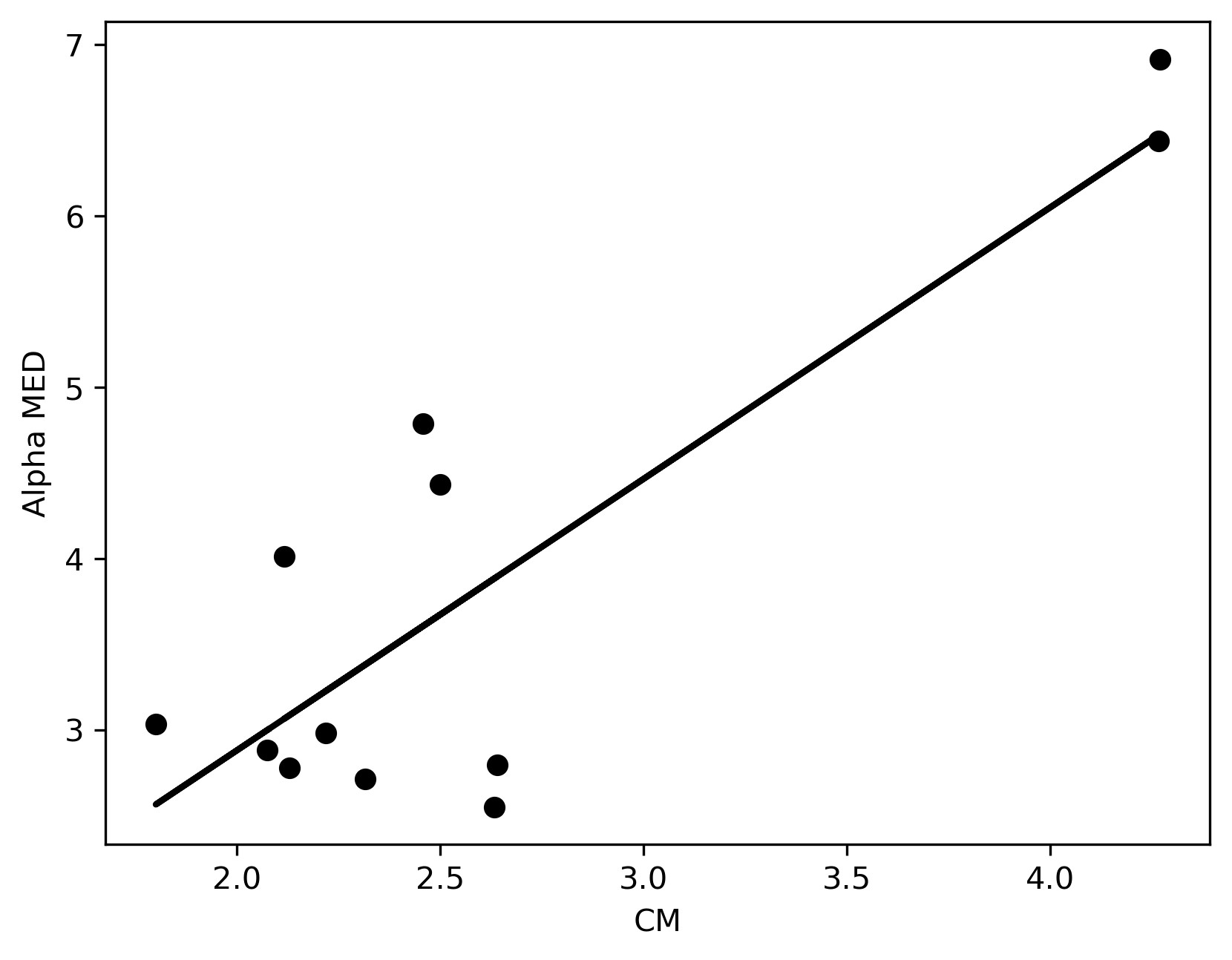

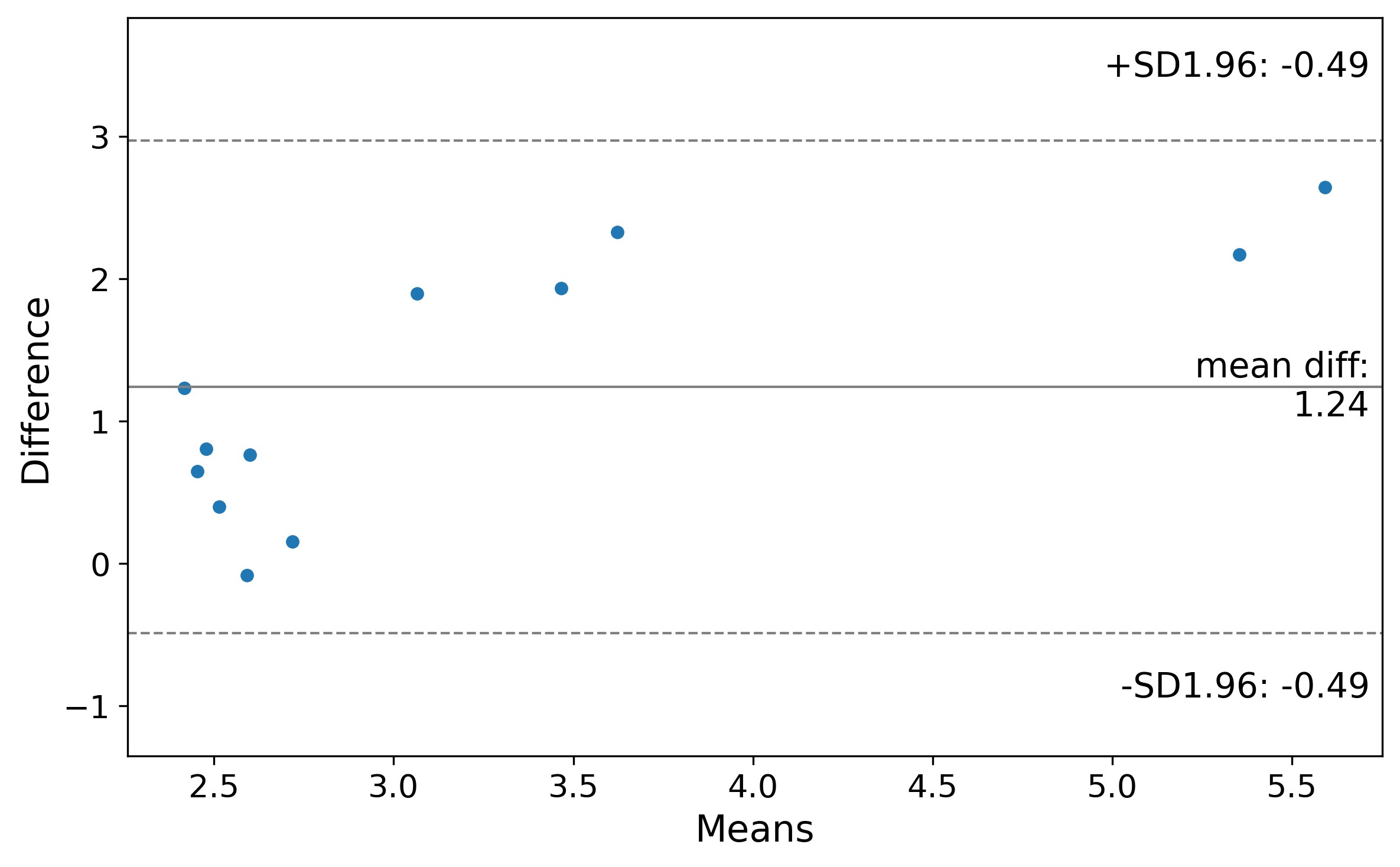


**Supplementary Figure 3.** Bland-Altman and regression plot. On the left: Linear regression between the CM and Alpha MED method. On the right: Bland-Altman plot between the CM and Alpha MED digital method.


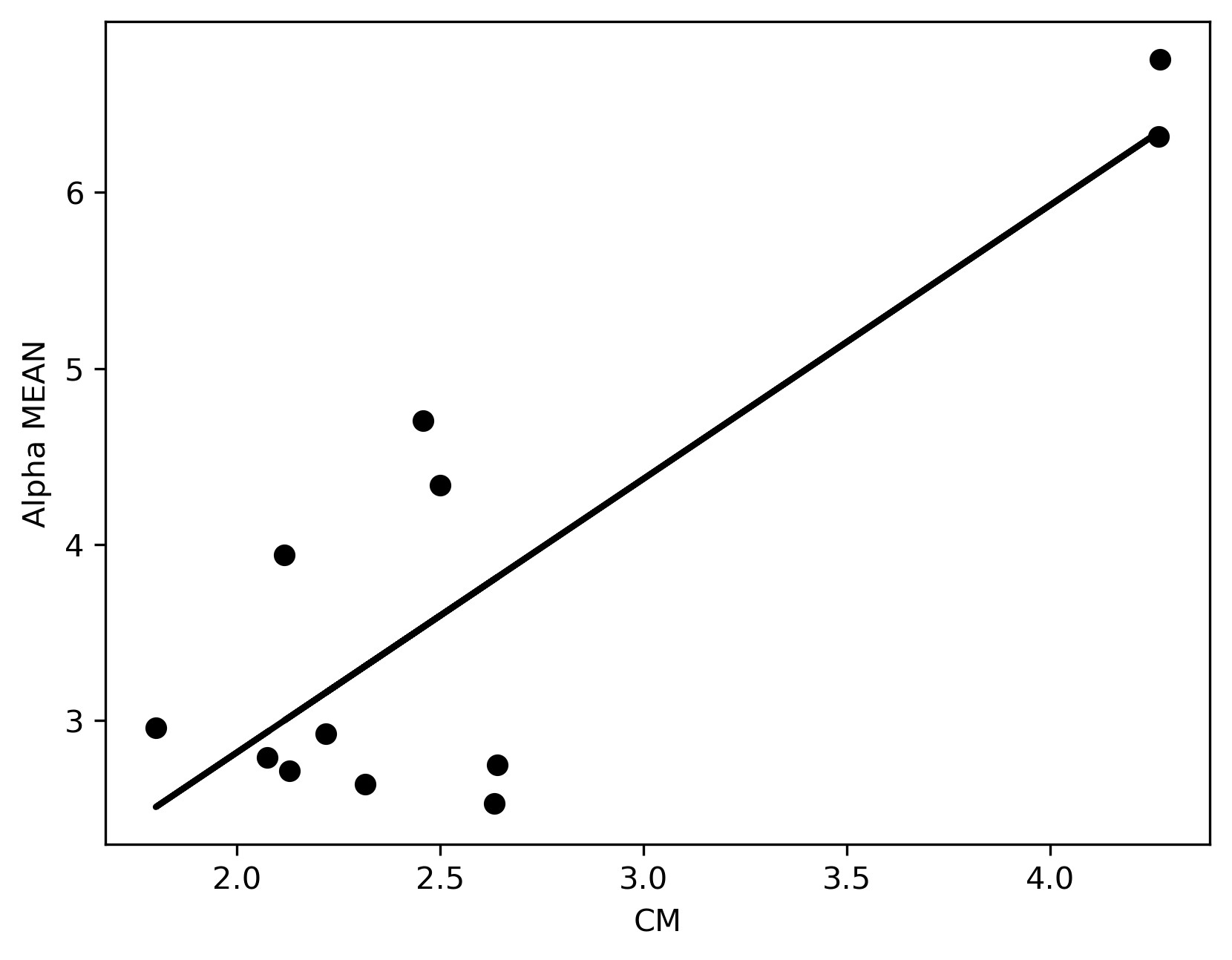

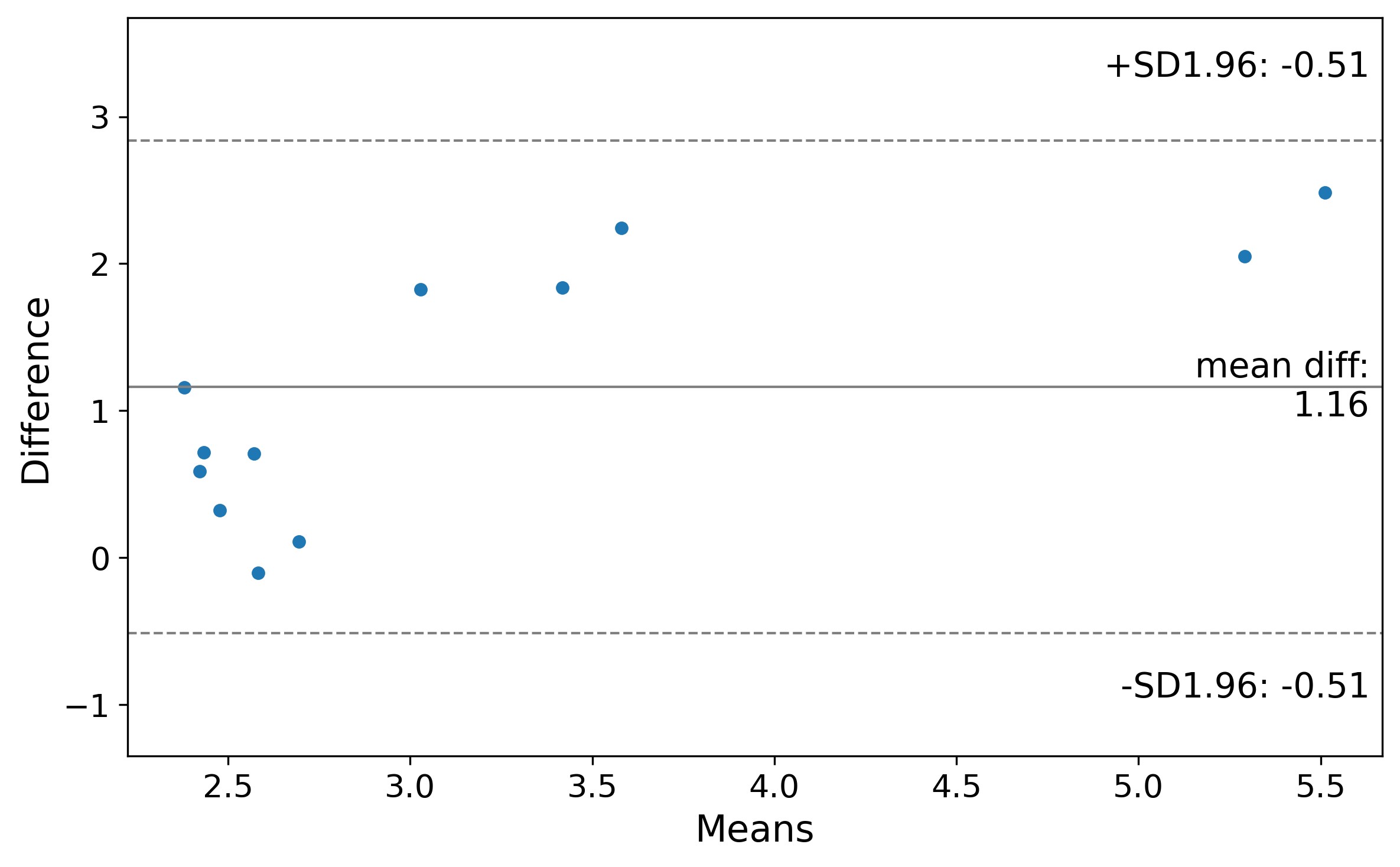


**Supplementary Figure 4.** Bland-Altman and regression plot. On the left: Linear regression between the CM and Alpha MEAN method. On the right: Bland-Altman plot between the CM and Alpha MEAN digital method.


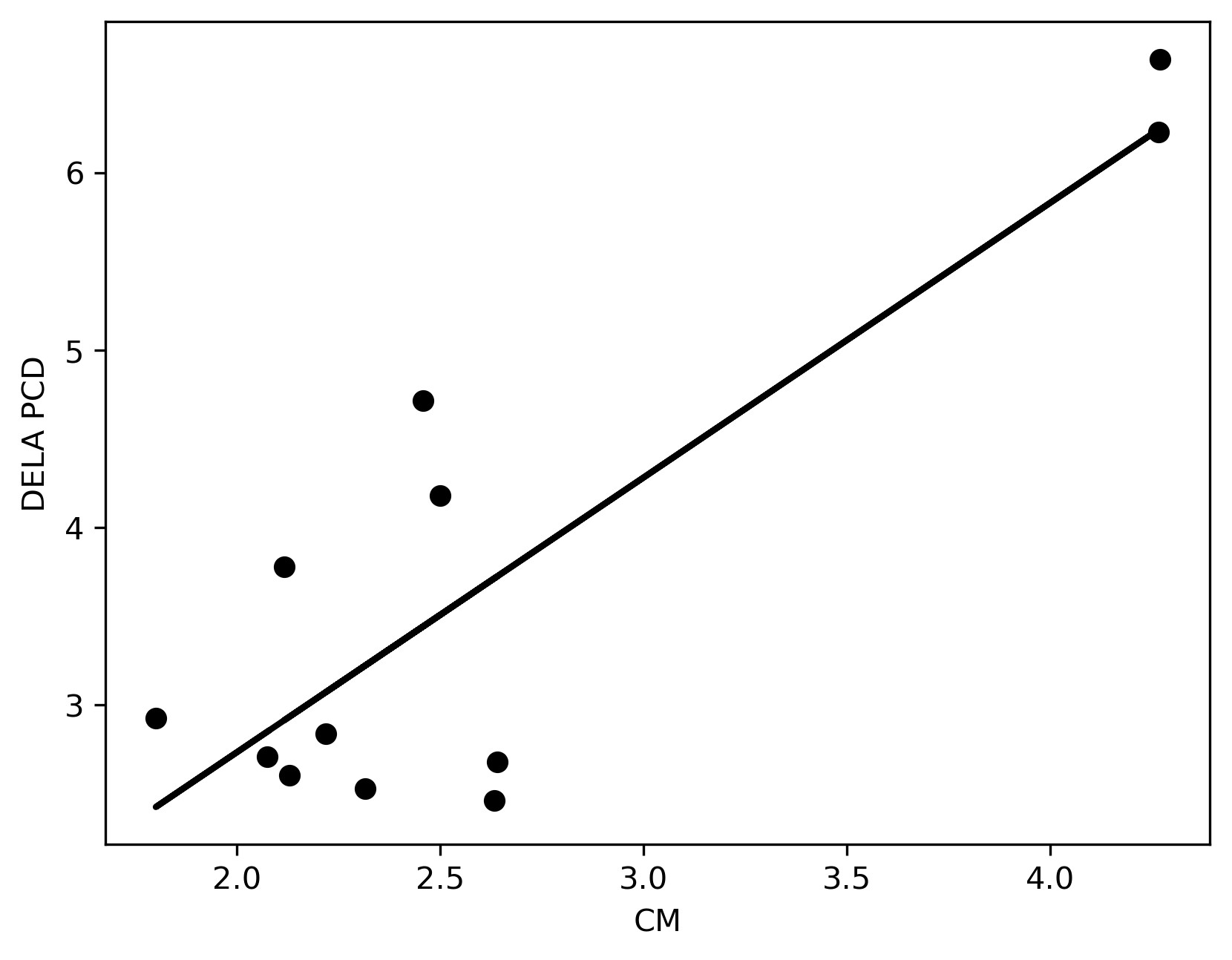

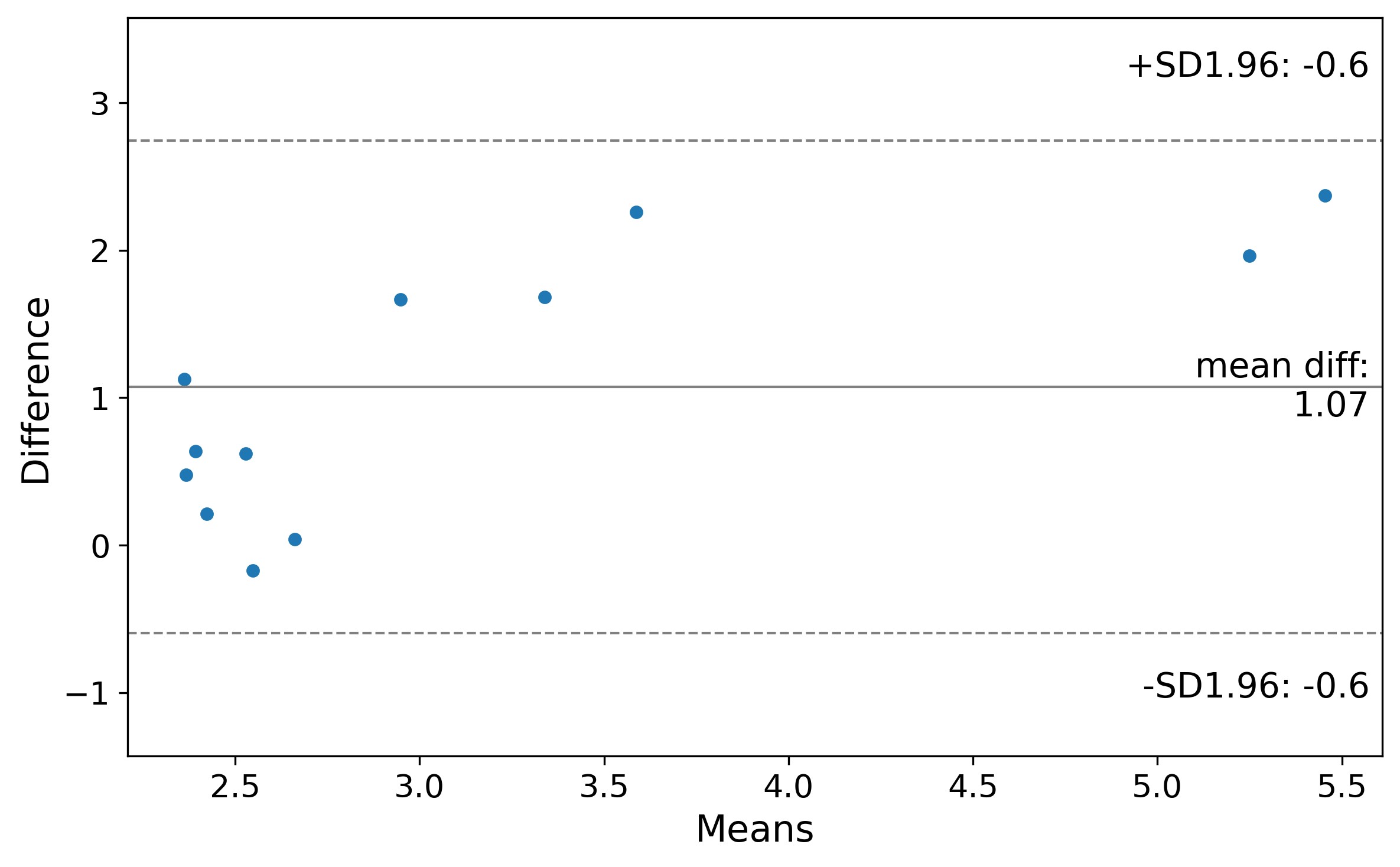


**Supplementary Figure 5.** Bland-Altman and regression plot. On the left: Linear regression between the CM and DELA PCD method. On the right: Bland-Altman plot between the CM and DELA PCD digital method.
